# Surgical Versus Conservative Management of Acute Appendicitis During the COVID-19 Pandemic: A Single-Centre Retrospective Study

**DOI:** 10.7759/cureus.14095

**Published:** 2021-03-24

**Authors:** Andrew Lotfallah, Amaar Aamery, George Moussa, Mangta Manu

**Affiliations:** 1 Surgery, New Cross Hospital, Wolverhampton, GBR; 2 General Surgery, New Cross Hospital, Wolverhampton, GBR; 3 Ophthalmology, Birmingham Midland Eye Centre, Birmingham, GBR

**Keywords:** appendicitis, appendicectomy, surgery, conservative, covid-19, coronavirus, management

## Abstract

Introduction

The COVID-19 pandemic provoked a change to normal surgical practice in the United Kingdom and led to an increase in acute appendicitis (AA) patients being treated conservatively with antibiotics. We aim to analyse the management of patients presenting with AA to our institution during the first wave of the pandemic, comparing surgically and conservatively managed patients.

Method

All patients presenting to our centre with AA between March and July 2020 were included. Six-month follow-up data were collected retrospectively using electronic records. Patients were categorised into surgically and conservatively managed groups. The primary outcome was the complication rate (post-operative complications vs failure of antibiotic treatment) and the secondary outcomes were length of hospital stay and Alvarado score.

Results

Fifty-seven patients (n=57) were admitted with AA, 45.6% (n=26) managed conservatively compared to 54.4% (n=31) treated surgically. Higher complication rates were observed amongst the conservatively managed group, although not found to be statistically significant (16% vs 35%; p=0.131). There was no significant difference in length of hospital stay observed between the two groups (surgical: median, 2; interquartile range, 2-3 vs conservative: median, 3; interquartile range, 2-4). White cell count (WCC) and Alvarado score were higher on admission in the surgical group with statistical significance (p=0.012 and p=0.028, respectively).

Conclusions

COVID-19 has led to a significant cohort of conservatively managed AA patients in the United Kingdom. We propose a stratification pathway based on clinical severity, Alvarado score and imaging to facilitate safe selection for conservative management of AA, in order to reduce failure of treatment rates in this patient group. Further UK-based studies will add to the evidence-based surrounding safe management of AA with conservative treatment.

## Introduction

Acute appendicitis (AA) is the commonest general surgical emergency worldwide, with a lifetime risk of 7%-8% [1,2]. Appendicectomy has been considered the first-line treatment for AA for over a century, with approximately 50,000 performed in the United Kingdom annually [3]. The procedure has become routine and is associated with a significantly low mortality [4]. Early appendicectomy is recommended to avoid serious complications of appendicitis such as perforation, abscess formation and faecal peritonitis [5]. However, as with any surgical intervention, appendicectomy still involves significant risks [4].

A growing evidence-base suggests that conservative treatment with antibiotics is an effective management strategy for cases of non-complicated appendicitis [6-8]. In the last decade, multiple meta-analysis and systematic reviews have been conducted comparing surgical and conservative management of AA [7-9]. The majority concludes that whilst appendicectomy remains the definitive treatment option, antibiotic treatment is a safe and effective alternative.

The impact of COVID-19 has made the choice between surgical and conservative AA management significantly more pertinent. The pandemic has brought about widespread concern over viral spread caused by aerosol-generating procedures. Furthermore, surgical staff mobilisation to critical care and medical ward settings to manage the overwhelming pressures of COVID-19 has resulted in reduced surgical capacity. Following the initial outbreak of the pandemic, the Royal College of Surgeons of England (RCSEng) issued nation-wide guidance to minimise surgery where possible in favour of conservative management [10]. Consequently, patients presenting with AA who likely otherwise would have been managed surgically, were treated conservatively during the first wave of the pandemic throughout the United Kingdom. Analysis of this patient cohort can further expand on previous studies comparing the use of antibiotics to surgery to treat AA. This study aimed to compare short-term complication rates between patients managed conservatively and surgically during the first wave of COVID-19 in our institution. Length of hospital stay and initial Alvarado score were compared as secondary outcomes. It is hoped that the findings of this study will lead to further implications and recommendations for clinicians treating AA conservatively as we continue to face the pandemic and beyond. 

A summary of this study was presented as an online poster at the ASiT Annual Conference 2021 on 6-7th March, 2021.

## Materials and methods

This is a retrospective continuous comparative study of all patients admitted to our district general hospital between 1st March and 1st July 2020 with AA. A database of patients admitted to our institution clinically coded as “Acute appendicitis” on discharge summaries in the given time period was generated. Data on each patient was collated retrospectively using the Trust electronic patient web portal, which included demographic details, co-morbidities, inflammatory markers on admission and imaging undertaken. Diagnosis of AA was made on clinical grounds by the admitting consultant - based on history, positive examination findings (tenderness and guarding in the right iliac fossa) and raised inflammatory markers with a negative urinalysis (and negative pregnancy test in female patients). The management strategy for each patient was recorded (surgical vs conservative) as well as length of hospital stay. Six-month follow-up data from electronic records were acquired retrospectively by analysing outpatient letters, emergency department (ED) attendances and readmissions.

Failure of antibiotic treatment in the conservative group was defined as: appendectomy in the follow-up period (during same admission or on subsequent admission); recurrence of right iliac fossa (RIF) pain; re-admission with RIF pain. Post-operative complications in the surgical group were classified as procedure-specific complications (wound or surgical site infection, adhesions, port-site or incisional hernia and/or persistent surgical site pain four weeks after discharge) and general complications (e.g., pneumonia, venous thromboembolism, urinary tract infection etc.).

Importantly to note, given the rapid changes to practice that were made during the initial outbreak of COVID-19 in the United Kingdom, no selection criteria or guidelines were formulated within our institution to determine whether patients were more suited to surgical or conservative management. Management strategy (conservative vs surgical) was decided on by the admitting surgical consultant on a case-by-case individual clinical assessment. Regarding antibiotic therapy, our surgical firm makes use of local trust antibiotic guidelines which recommend triple intravenous therapy (amoxicillin (or teicoplanin in penicillin-allergic patients), metronidazole and gentamicin) to treat non-complicated cases of AA.

Criteria for safe discharge used were as follows: pain improving and manageable in the community; downward trend in white cell count (WCC); apyrexial (temperature < 37.8 degrees Celsius) for more than 24 hours. Social factors, such as care needs and independence, may also limit the point at which a patient is discharged.

Statistical analysis

Statistical significance was defined as p<0.05. Prior to analysis, normality of continuous variables was assessed using the Shapiro-Wilk test, and found not to be normally distributed. Hence, continuous data are primarily reported as medians and interquartile ranges (IQRs) throughout. Mann Whitney U was used to compare two continuous variables. Fisher exact test was used to compare two nominal variables. All statistical analysis was performed using IBM SPSS Statistics for Windows, Version 26.0 (IBM Corp, Armonk, NY) and Microsoft Excel 365.

## Results

An initial dataset of 64 patients was provided by the Trust Information Department for analysis. Four patients were excluded due to incorrect clinical coding and a further three were excluded due to insufficient clinical information on the clinical web portal. A total of 57 patients were included for final analysis with a diagnosis of AA between 1st March 2020 to 1st July 2020 (median age 25 [IQR 18-43], 67% male). A summary of baseline clinical characteristics is reported in Table 1.

**Table 1:** Baseline clinical characteristics of patients treated with acute appendicitis. -Continuous variables are reported as median (interquartile range). -Mann-Whitney U-test was used to compare continuous variables between groups. -Percentages are reported to one decimal place. -p-values reported to three decimal places (statistical significance in bold). CT: computerised tomography; USS: ultrasound scan; AA: acute appendicitis; WCC: White cell count; CRP: c-reactive protein.

	Total	Surgical	Conservative	p-value
n	57	31	26	-
Age	25 (18-43)	24 (12-43)	29.5 (22.25-48.25)	-
Gender (% male)	38 (66.7%)	22 (71.0%)	16 (61.5%)	
Imaging				
USS	21 (36.8%)	8	13	-
CT	17 (29.8%)	8	9	-
Both (CT and USS)	1 (1.8%)	1	0	-
Diagnosis based on imaging				
Nil acute	8 (14.0%)	2	6	-
Uncomplicated AA	23 (40.4%)	11	12	-
Complicated AA	5 (8.8%)	4	1	-
Inconclusive	3 (5.3%)	0	3	
No imaging	18 (31.6%)	14	4	-
Primary surgery				
Converted to open	1 (3.2%)		-	-
Laparoscopic	19 (61.3%)		-	-
Open	11 (35.5%)		-	-
Inflammatory markers				
WCC	13.6 (10.6-16.5)	15.9 (12.2-17.4)	12.5 (10.4-15.6)	0.012
CRP	31.0 (7.0-104.0)	50.0 (17.0-114.0)	24.5 (6.0-99.0)	0.246
Alvarado score	7 (6-8)	8 (6-9)	6 (5-8)	0.028

There were 31 patients (54.4%) that were treated surgically with appendicectomy (laparoscopic - 19; open - 11; laparoscopic converted to open - 1), and 26 (45.6%) patients were treated conservatively with intravenous antibiotics only.

Complications at six-month follow-up were more frequent in the conservatively managed group compared to the surgically managed group at six-month follow-up; however, statistical difference was not demonstrated (conservative: n=9, 34.6%, surgical: n=4, 12.9%, p=0.064) (Table 2). No significant difference was found in length of hospital stay between the two groups (surgical: median 2 [IQR 2-3], conservative: median 3 [IQR 2-4]). WCC (surgical: median 15.9 [IQR 12.2-17.4], conservative: median 12.5 [IQR 10.4-15.6]) and Alvarado score (surgical: median 8 [IQR6-9], conservative: median 6 [5-8]) on admission was significantly higher in the surgical group compared to the conservatively managed group (p=0.012 and p=0.028, respectively). A summary of all patients having complications within six-month follow-up in both groups is provided in Table 3, including imaging undertaken and initial WCC and Alvarado score.

**Table 2:** Length of hospital stay and complications breakdown. -Length of stay is reported as median (interquartile range). - Mann-Whitney U-test was used to compare continuous variables between groups. -Fisher-exact test (two groups) was used to compare nominal groups. -Percentages are reported to one decimal place. -p-values reported to three decimal places. *Presenting to Emergency Department/Outpatient clinic but not readmitted.

	Total	Surgical	Conservative	p-value
n	57	31	26	-
Length of hospital stay	2 (2-3)	2 (2-3)	3 (2-4)	0.863
Complications (% yes)				-
Any complication	13(22.8%)	4	9	0.064
Further Surgery	8 (14.0%)	2	6	-
Performed	7 (12.3%)	2	5	-
Planned	1 (1.8%)	0	1	-
Readmission	10 (17.5%)	4	6	0.486
Emergency readmission	9 (15.8%)	4	5	-
Elective readmission	1 (1.8%)	0	1	-
Perforation	2 (3.5%)	0	2	-
Recurrence of symptoms*	4 (7.0%)	1	3	0.322

**Table 3:** Summary of cases involving complications within six-month follow-up. CT: computerised tomography; USS: ultrasound scan; AA: acute appendicitis; RIF: right iliac fossa.

Surgery	Case no.	Gender	Age	Imaging	Findings on imaging	White cell count	Alvarado score	Complication	Further surgery (findings)
	1	M	74	CT	Contained perforated appendix	20.1	6	Adhesional small bowel obstruction	Laparotomy
	2	F	52	USS & CT	USS inconclusive, CT typical AA	12.2	8	Surgical site pain >4 weeks post-discharge	-
	3	M	9	USS	Typical AA	16.3	8	Surgical site pain >4 weeks post-discharge	-
	4	M	27	None	n/a	13.6	8	Stump appendicitis	Completion appendicectomy
Conservative	Case no.	Gender	Age	Imaging	Finding on imaging	White cell count	Alvarado score	Complication	Further surgery (findings)
	1	M	58	CT	Typical AA	4.9	5	Emergency appendicectomy	Laparotomy - perforated appendix
	2	M	28	None	n/a	14.6	8	Recurrence of RIF pain	-
	3	F	53	CT	Typical AA	7.6	3	Recurrence of RIF pain	Consented for elective appendicectomy
	4	M	24	None	n/a	10.4	6	Readmitted with RIF pain	-
	5	F	26	CT	Mildly distended appendix	15.4	5	Emergency appendicectomy	Inflamed appendix, no perforation
	6	M	19	None	n/a	25.9	6	Emergency appendicectomy	Inflamed appendix, no perforation
	7	F	43	CT	Typical AA	9.6	4	Recurrence of RIF pain	-
	8	F	9	USS	Possible appendiceal mass	12	4	Recurrence of RIF pain	Elective appendicectomy
	9	F	34	USS - inconclusive	Appendix obscured by bowel (inconclusive)	19.6	9	Emergency appendicectomy	Perforated appendix

Computerised tomography (CT) scan was carried out in 18 cases and ultrasound scan (USS) in 22 (Table 1). Uncomplicated AA was the diagnosis on imaging in 23 cases, 11 of which were managed surgically and 12 conservatively. Complicated AA (associated abscess, collection, perforation and/or peritonitis) was diagnosed on imaging in five cases, four of which were managed surgically and one managed conservatively. The one conservatively managed case had AA with a small collection on CT and did not suffer complications at six months. Inconclusive scans (all of which were USS) were reported in three cases, all of which were managed conservatively. No imaging was carried out in 18 cases, 14 of which were managed surgically.

Of the nine conservatively managed patients who suffered complications at six-month follow-up, four had initial CT scan, two had USS, one of which was inconclusive, and the three remaining had no imaging (Table 3). Of the four patients managed surgically who had post-operative complications, one patient had CT scan confirming perforated appendicitis, one patient had an inconclusive USS followed by CT revealing uncomplicated AA, USS only was performed in one case and no imaging in one (Table 3).

## Discussion

The COVID-19 pandemic has placed immense pressures on our healthcare system. The field of surgery has made fundamental changes to adapt during this uncertain time, prioritising emergency surgery and attempting to minimise the spread of the virus by suspending elective procedures. At the beginning of the first wave of COVID-19, the Royal College of Surgeons of England (RCSEng) published nationwide guidance which included the recommendation to seek “Non-surgical solutions (…) to avoid surgery where possible” [10]. As such, conservative management with antibiotics has been more widely used to treat cases of AA during the pandemic nationally. The HAREM study investigating 500 patients from 48 sites treated for appendicitis during a similar catchment period to the current study found that 54% (n=271) were treated conservatively [11].

Conservative management was more widely implemented to treat AA in our institution following the onset of COVID-19 in accordance with national guidelines [10]. Decision to treat non-operatively was made on a case-by-case basis by the admitting surgical consultant without any formalised stratification criteria or pathway to guide choice between conservative and surgical management. This may explain the higher rate of complications observed in the conservatively managed group, with four requiring emergency appendicectomy in the six-month follow-up period, two of which were found to have a perforated appendix at operation. 

We found no significant difference in length of hospital stay between the two groups (surgical: median 2 IQR 2-3; conservative: median 2 IQR 2-4). Median length of stay was similarly reported in the APPAC randomised clinical trial involving 529 patients (surgical: median 3 IQR 2-3; conservative: median 3 IQR 3-3) [12]. This indicates no notable disadvantage regarding length of hospital stay when comparing conservative and surgical management of AA, an important consideration in terms of patient outcome and experience. It also suggests that the use of antibiotic treatment alone does not significantly prolong bed occupancy. This is of particular importance in the current COVID-19 pandemic as hospitals have been stretched to full capacity to facilitate the treatment of COVID-19 patients.

Managing AA with antibiotics is by no means a novel concept. Successful antibiotic treatment of AA was formally reported by Harrison in 1953 and Coldrey in 1959 [13,14]. In the last decade, several large-scale systematic reviews and meta-analyses have compared the use of antibiotics to conservatively manage AA with surgical appendicectomy [7-9]. A systematic review of five randomised control trials involving 1430 patients, showed no significant difference between length of hospital stay and probability of complication-free treatment, comparing conservatively and surgically managed AA [7]. This was in keeping with the findings of the current study (length of stay: p=0.863, complications: p=0.064). Although there was no significant difference in complication rate in the aforementioned systematic review, 37.4% (n=727) of conservatively managed patients went on to have appendicectomy at one year. The study, therefore, concluded that appendicectomy was more effective than antibiotic therapy as a definitive treatment [7]. This is in comparison to a multicentre randomised clinical trial involving 529 patients which demonstrated 27.3% of patients requiring appendicectomy at one year following initial conservative therapy [12]. Our analysis at six-month follow-up has revealed similar results, with 23% (n=6) patients undergoing appendicectomy (performed n=5, planned n=1) in the conservatively managed group. Whilst appendicectomy remains the definitive curative treatment option, antibiotics are considered a safe alternative in uncomplicated AA, despite the known risk of recurrence and subsequent operation [7,8,12].

The identification of non-complicated cases of AA is a crucially important step in the decision to treat conservatively. Complicated AA is defined as involving perforation, peritonitis, abscess and/or empyema formation [7,8]. Whilst AA is diagnosed clinically, CT scan can be reliably used to distinguish between complicated and non-complicated cases [14]. The APPAC study advocates the routine use of CT scan to establish non-complicated cases of AA and reduce the rate of negative appendicectomy [14]. According to a worldwide prospective observational study involving 4282 patients with AA, CT scan was performed in a third of cases [15]. This finding was corroborated by the present study, in which 31.6% (n=18) of patients analysed had a CT scan.

Of the 18 patients that had no imaging, the majority were treated surgically (n=14) compared to those treated conservatively (n=4). This may indicate that clinicians were more likely to opt for surgical management to treat AA without the benefit of definitive imaging to exclude complicated cases. Only 29.0% (n=9) of all surgically managed patients underwent definitive imaging with CT scan prior to surgery. Had CT imaging been undertaken more frequently, more patients may have been selected for conservative management, reducing both general and COVID-19 related risks associated with surgery.

Notably, four conservatively managed patients underwent emergency appendicectomy within the six-month follow-up period, two of which had no imaging and one who had an inconclusive ultrasound scan due to bowel obscuring the appendix on primary admission. The latter patient subsequently underwent laparoscopic appendicectomy which revealed a perforated appendix. This particular case demonstrates the importance of definitive imaging prior to selection for conservative management, and the limitations of USS compared to CT.

The Alvarado scoring system can be used in the assessment of AA as a useful prognostic indicator [16]. However, evidence suggests that it cannot be relied upon in isolation to identify patients suitable for conservative management. The NOTA study found a failure rate (readmission within seven days due to lack of clinical improvement or worsening symptoms) of 11.9% in patients initially deemed suitable for conservative management based primarily on Alvarado score (5-6) [17]. According to a systematic review, Alvarado score performs more favourably as a ‘ruling out’ score with 5 as a cut-off point, with an overall sensitivity of 99% [18]. However, with an overall specificity of 81%, it performs less well as a ‘ruling in’ score [17]. We identified that Alvarado score was significantly higher in the surgical cohort (p=0.028). This finding is consistent with the selection for surgery being based on clinical assessment involving the same parameters used to determine the Alvarado score. This suggests that in the significant number of cases, more severe cases of AA were treated operatively.

The Alvarado score is thus a useful indicator of patients more likely to require surgery but best used in conjunction with sound clinical judgement and imaging to determine the severity of AA and need for surgery, rather than as an isolated determinant. USS in patients with low Alvarado score can mitigate the possibility of missed complicated cases.

Proposed stratification tool

**Figure 1:**
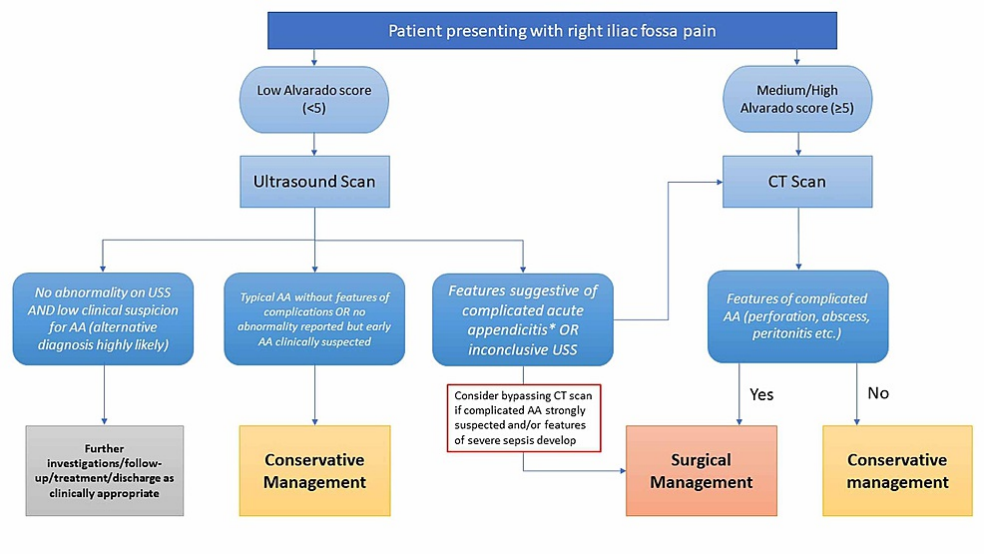
Stratification pathway for acute appendicitis management. *Features of complicated AA – abscess or empyema formation, perforation and/or peritonitis. AA: acute appendicitis.

Based on our findings, we have proposed a stratification pathway (Figure 1) to guide decision-making relating to conservative AA management within our surgical department. Patients presenting with low Alvarado score (<5) should undergo USS as a preliminary imaging. If the USS suggests features of complicated appendicitis or is inconclusive, the patient should proceed to definitive imaging with CT scan. Alternatively, if there is sufficient clinical evidence of complicated appendicitis supported by findings on USS, the clinician may choose to proceed straight to surgical management without CT. If the Alvarado score is low and the USS shows typical AA, the patient can be stratified into the conservative management group with greater clinical confidence. If, however, no abnormality is detected on USS and an alternative diagnosis to AA is more likely based on clinical assessment, the clinician should pursue alternative diagnostic and management pathways.

Should the patient present with medium to high Alvarado score (≥5), then definitive imaging with CT scan is recommended. The management of patients proceeding to CT scan (surgical vs conservative) will be determined by the absence or presence of complicated AA.

Limitations

The authors recognise the limitations of this study. Patients were not randomised into surgical and conservatively managed groups. As a result of the rapid adaptations which had to be made in response to the pandemic, management strategies were based on the assessment and clinical judgement of the individual consultant which will inevitably have introduced selection bias. Furthermore, the choice of antibiotic regimen and duration of intravenous and oral antibiotic treatment was variable and again, based on the discretion of the admitting clinician. Moreover, the results and recommendations of the current study are based on a relatively short-term follow-up of six months and small sample size.

## Conclusions

The use of conservative management to treat AA compared to appendicectomy remains an area of dispute. However, with careful selection criteria, conservative management of AA can be both safe and effective. As the pressures of the COVID-19 pandemic begin to ease following the implementation of successful vaccination rollout nationally, it is important for UK-based centres to share their experience of managing AA. Whilst AA remains a clinical diagnosis, we have recommended a stratification pathway to facilitate safe patient selection for conservative management of AA. This will be relevant in situations where non-surgical management is being considered or preferred, as was the case during the initial phase of the pandemic. It is hoped that the guidance provided by this stratification pathway will result in fewer complications amongst conservatively managed patients and reduce the rate of negative appendicectomies. Analysis of this stratification pathway following implementation will seek to evaluate its effectiveness. In addition, further UK-based studies surrounding AA management during COVID-19 will add to the evidence base comparing conservative and surgical management of AA.
